# Physiological relaxation induced by horticultural activity: transplanting work using flowering plants

**DOI:** 10.1186/1880-6805-32-15

**Published:** 2013-10-10

**Authors:** Min-sun Lee, Bum-jin Park, Juyoung Lee, Kun-tae Park, Ja-hyeong Ku, Jun-woo Lee, Kyung-ok Oh, Yoshifumi Miyazaki

**Affiliations:** 1 Center for Environment, Health and Field Sciences, Chiba University, Kashiwa, Japan; 2 Department of Environment and Forest Resources, Chungnam National University, Daejeon, Korea; 3 Korea Forest Service, Government Complex 1, Daejeon, Korea; 4 Department of Horticulture, Chungnam National University, Daejeon, Korea; 5 Department of Nursing, Chungnam National University, Daejeon, Korea

**Keywords:** Horticultural activity, Therapeutic effects, Heart rate variability, Autonomic nervous activity, Personality difference

## Abstract

**Background:**

Despite increasing attention and a growing volume of research data, little physiological evidence is available on the benefits of horticultural activity and the different effects on individuals. Therefore, the aim of the present study was to investigate the physiological effects of horticultural activity and to examine how differences in personality alter these effects.

**Results:**

The effects of transplanting real flowers (horticultural activity) and handling artificial flowers (control activity) on human physiological activity were compared. On the first day, eight participants engaged in horticultural activity and another eight in the control activity. On the second day, participants switched roles. Participants’ physiological conditions during each activity were assessed by measuring the heart rate and heart rate variability (HRV). Psychological responses, which were measured using a semantic differential rating scale, showed that the horticultural activity promoted comfortable, soothed, and natural feelings, compared to the control activity. Analysis of physiological responses using two-way repeated measures analysis of variance (ANOVA) revealed that sympathetic nervous activity significantly decreased in the late time period (11 to 15 minutes) of horticultural activity only in the type A group.

**Conclusions:**

This study supports the fact that the horticultural activity can enhance psychological and physiological relaxation effects, although these physiological effects can differ among individuals with different personalities.

## Introduction

Humans have spent most of their evolution in natural environments [1] and have an innate desire to interact with such environments (biophilia hypothesis) [2]. The natural environment plays an important role in health promotion, and theoretical foundations for the positive relationships between human health and natural environments have been developed. The attention recovery theory [3,4] and stress reduction theory [5] support that natural environments effectively facilitate recovery from attentional fatigue and psychological stress.

Increasing attention has been paid to the beneficial effects of nature-related activities, including gardening or horticultural activity, which is considered a health promoter [6]. To date, various studies have demonstrated that gardening or horticultural activities reduce stress [3] and improve self-esteem, social interactions [7], and cognitive health [8]. Due to these beneficial effects, horticultural activity has often been used in rehabilitation programs for patients with different types of disorder [9,10]. It has also been reported that flower decoration, one such typical horticultural activity, can ease negative feelings and enhance cognitive ability [11]. Despite many studies on this subject, little scientific evidence is available on the physiological effects of horticultural activity.

In addition, human responses to external stimuli differ depending on an individual’s personality; that is, the important and relatively stable characteristics of a person that account for consistent patterns of behavior [12]. So far, most personality studies have been performed by observing external and social behavior; however, recent reports in the field of physiological anthropology support the idea that personality can also be studied by investigating physiological response patterns. Previous experiments have illustrated that human physiological responses to natural stimuli differ according to personality [13,14], indicating the importance of personality studies from the perspective of physiological anthropology. Despite this importance, there are only a few studies that have verified individual differences based on personality in the physiological effects of horticultural activity. Therefore, we examined the differences in the physiological effects among individuals with different personality traits, characterized by a type A and type B behavior pattern [15,16].

The aim of this study was to investigate the psychological and physiological effects of horticultural activity and examine how differences in personality alter these effects from the viewpoint of physiological anthropology.

## Methods

### Participants

The study participants were 16 Korean male university students with an age (mean ± SD) of 26 ± 2.1 years who were non-smokers and had no history of physical or psychological disorders. Alcohol, tobacco, and caffeine intake were prohibited throughout the experimental periods. Prior to the start of experiments, a full explanation was provided on this study, and participants’ informed consent was obtained. This study was conducted following the regulations of the Clinical Trials Center, Chungnam National University Hospital, Korea, and the Ethics Committee of the Center for Environment, Health, and Field Sciences, Chiba University, Japan.

## Materials

**Figure 1:**
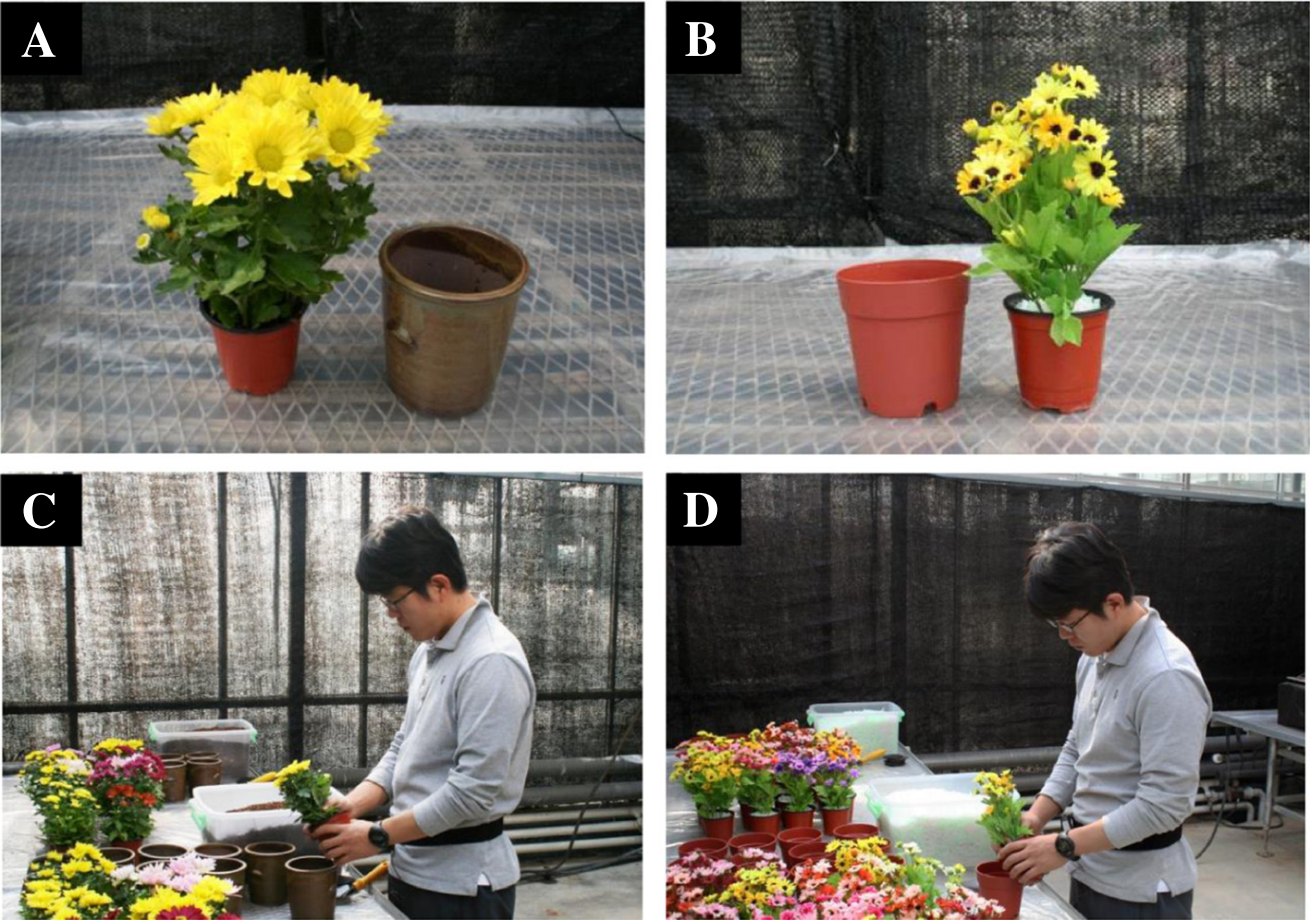
**Photographs of materials used in experiments. (A)** Real flower (*Chrysanthemum*); **(B)** artificial flower; **(C)** a subject doing horticultural activity; **(D)** a subject doing control activity.

As a typical horticultural activity, a transplanting activity using real soil and flowers (*Chrysanthemum morifolium*) was selected. The transplanting method was taught to each participant prior to experiments. The materials used in this study are shown in Figure 1. Transplanting of artificial flowers of almost the same size and weight as real flowers was performed as a control activity. Plastic scraps (approximately 2 to 5 mm in diameter) instead of soil were used for the control activity. Horticultural and control activities were carried out on the planting bed in a standing position for 15 minutes (Figure 1).

### Protocol

Participants were randomly divided into two groups. On the first day of experiments, the first group (N = 8) carried out horticultural activity, while the second group (N = 8) performed the control activity. On the second day, each group switched activities as a crosscheck.

### Measurements

Each participant was guided to the experimental room and rested in a seated position for 2 minutes. Then, the participant performed horticultural or control activities for 15 minutes individually. During this activity, heart rate variability (HRV) was continuously measured using a portable electrocardiograph (Activtracer AC-301A; GMS, Tokyo, Japan). After horticultural or control activities, participants rested in a seated position for 2 minutes, followed by subjective evaluation using questionnaires. HRV data were analyzed with maximum entropy methods (MemCalc, GMS, Tokyo, Japan) where low (LF: 0.04 to 0.15 Hz) and high frequency (HF: 0.15 to 0.40 Hz) components of the power spectrum were calculated based on the R-R interval [17]. The HF component of HRV was used as an index of parasympathetic nervous activity, which increases when in a relaxed state, and the LF/(LF + HF) value of HRV as an index of sympathetic nervous activity, which increases when in a stressed state [18,19]. All HRV values were logarized. HRV data were analyzed across three periods of time: early (1 to 5 minutes), middle (6 to 10 minutes), and late (11 to 15 minutes). Self-report-type questionnaires were administered using a seven-scaled semantic differential (SD) method for the feelings of 'comfortable’, 'soothing’, and 'natural’.

### Personality

The type A behavior pattern was used to classify personal characteristics, which is one of the most important factors regulating differences in cardiac responses [20-22]. The type A behavior patterns were identified using a brief questionnaire [23] and participants were categorized into two groups: type A (N = 8) and type B (N = 8).

### Statistical analysis

Two-way repeated measures analysis of variance (ANOVA) was used for the analysis of physiological data, followed by a simple main effect test for the post hoc analysis. The Wilcoxon signed-rank test was performed to verify the statistical differences in the psychological responses. SPSS 21.0 (SPSS Inc., Chicago, IL, USA) was used for statistical analysis, and the differences were considered significant at *P* <0.05.

## Results

**Figure 2:**
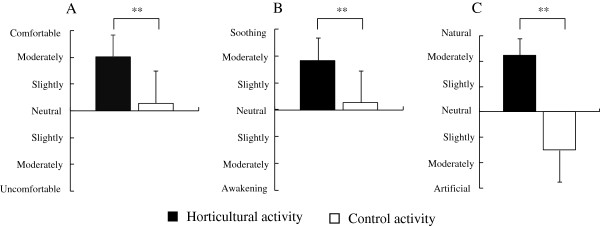
**Comparisons of psychological assessment after horticultural and control activities. (A)** Feeling comfortable; **(B)** feeling soothed; **(C)** feeling natural. N = 16, mean ± SD, **P <0.01 (Wilcoxon signed-rank test).

There were significant differences in the analysis of subjective evaluation between horticultural and control activities (Figure 2). After 15 minutes of activities, higher scores were seen for the feelings of 'comfortable’, 'soothing’, and 'natural’ in the horticultural activity than in that of the control.

**Figure 3:**
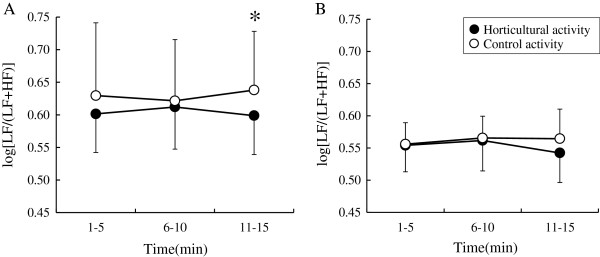
**Comparison of the values of log(LF/(LF + HF)) during a 15-minute horticultural and control activities using two-way repeated measures analysis of variance (ANOVA).** Mean ± SD. **(A)** Type A group (N = 8); *P <0.05; **(B)** type B group (N = 8); not significant. HF = high frequency, LF = low frequency.

There was no significant difference between horticultural and control activities in the mean values of heart rate, log(HF) and log(LF/(LF + HF)) for all subjects. However, the differences in the sympathetic nervous responses to two different activities (horticultural and control) were found in the type A group, but not in the type B group. For the type A group, the ANOVA of log(LF/(LF + HF)) tended to reveal an interaction effect (F _2,14_ = 3.188, *P* = 0.072) between the activities (horticultural and control) × time period (1 to 5, 6 to 10, and 11 to 15 minutes), and the simple main effect was yielded by the activities in the late time period (11 to 15 minutes; F _1,7_ = 5.848, *P* <0.05; Figure 3). However, the type B group revealed no interaction effect.

## Discussion

We examined the relaxing effects of a horticultural activity by measuring physiological and psychological changes based on the comparison of a transplanting activity using real and artificial flowers. Based on the subjective evaluation, participants felt more comfortable, soothed, and natural when transplanting real flowers compared to artificial flowers. The apparent psychological effects of horticultural activity are in part consistent with previous research related to natural stimuli [3,24-27].

Analysis of the physiological responses revealed differences in the effects of horticultural activities in the type A group. This group showed a significantly decreased sympathetic nervous response to the late time period of the horticultural activity compared to the control activity; however, the type B group revealed no significant changes. Decreased sympathetic nervous activity indicates that physiological tension or stress has eased, which is linked to physiological relaxation. The physiological data supports the fact that horticultural activity using natural materials can facilitate physiological relaxation by reducing sympathetic nervous activity [28], and these physiological benefits can differ among individuals depending on the type A personality [13,14,29]. This finding indicates that analysis of individual differences based on the type A personality might provide clues in considering physiological polymorphism, as reported in a previous study [13]. Although the reason why the physiological benefits occurred after 11 minutes of horticultural activity is not clear, this beneficial effect may be associated with nature-oriented stimulations including living flowers [30].

Despite the limitations in methodology including a lack of baseline data and a limited participant group, our findings provide insights for future research directions in related areas based on the perspective of physiological anthropology.

This study supports the fact that horticultural activity enhances not only the psychological state by increasing positive feelings but also physiological relaxation by reducing sympathetic nervous activity. In particular, the physiological benefits of horticultural activity can differ among individuals with different personalities.

## Competing interests

The authors declare that they have no competing interests.

## Authors’ contributions

ML participated in the study design, carried out data collection and analysis, and drafted the manuscript, BP participated in the study design and carried out data collection and interpretation, JyL participated in the study design and carried out data collection and analysis, KP carried out data collection and analysis, JK participated in the study design and data interpretation, JwL participated in study design and data interpretation, KO participated in data interpretation and helped to prepare the manuscript, and YM participated in the study design and data interpretation and improved the manuscript. All authors read and approved the final manuscript.
